# Automatic Fire Detection and Notification System Based on Improved YOLOv4 for the Blind and Visually Impaired

**DOI:** 10.3390/s22093307

**Published:** 2022-04-26

**Authors:** Mukhriddin Mukhiddinov, Akmalbek Bobomirzaevich Abdusalomov, Jinsoo Cho

**Affiliations:** 1 Department of Computer Engineering, Gachon University, Sujeong-gu, Seongnam-si 13120, Korea; mukhiddinov18@gachon.ac.kr; 2 Department of Artificial Intelligence, Tashkent University of Information Technologies Named after Muhammad Al-Khwarizmi, Tashkent 100200, Uzbekistan; bobomirzaevich@gmail.com

**Keywords:** fire detection, smart glasses, blind and visually impaired, assistive technologies, deep learning, object detection, CNN

## Abstract

The growing aging population suffers from high levels of vision and cognitive impairment, often resulting in a loss of independence. Such individuals must perform crucial everyday tasks such as cooking and heating with systems and devices designed for visually unimpaired individuals, which do not take into account the needs of persons with visual and cognitive impairment. Thus, the visually impaired persons using them run risks related to smoke and fire. In this paper, we propose a vision-based fire detection and notification system using smart glasses and deep learning models for blind and visually impaired (BVI) people. The system enables early detection of fires in indoor environments. To perform real-time fire detection and notification, the proposed system uses image brightness and a new convolutional neural network employing an improved YOLOv4 model with a convolutional block attention module. The h-swish activation function is used to reduce the running time and increase the robustness of YOLOv4. We adapt our previously developed smart glasses system to capture images and inform BVI people about fires and other surrounding objects through auditory messages. We create a large fire image dataset with indoor fire scenes to accurately detect fires. Furthermore, we develop an object mapping approach to provide BVI people with complete information about surrounding objects and to differentiate between hazardous and nonhazardous fires. The proposed system shows an improvement over other well-known approaches in all fire detection metrics such as precision, recall, and average precision.

## 1. Introduction

Disorders in the visual system can cause visual impairment and blindness, which may prevent individuals from performing housework as well as hindering their studies, work, travel, and participation in sports. According to the World Health Organization, at least 2.2 billion people worldwide suffer from visual impairment or blindness, of whom at least 1 billion have a visual impairment that could have been prevented or has not been addressed (as of 2020) [1]. Countries in South Asia and sub-Saharan Africa have the highest rates of visual impairment and blindness. Researchers predict that the number of individuals with visual impairment will increase dramatically in the next decades due to population growth and increasing life expectancy. Blind and visually impaired (BVI) individuals often find daily activities and environmental cognition (which refers to the awareness of one’s immediate surroundings) challenging. Several solutions such as assistive technologies and software are being developed to address such problems. Assistive systems aid BVI people with daily activities such as distinguishing banknotes [2], crossing a road [3,4], video media accessibility [5,6], image sonification for navigation [7], recognizing people [8], recognizing private visual information [9], selecting clothing [10], and navigating both outdoors and indoors [11,12].

For fire safety applications, assistive technologies have been developed to provide fire prevention and safety information to BVI individuals quickly during indoor and outdoor fire emergencies. However, these technologies suffer from some shortcomings.

**Figure 1:**
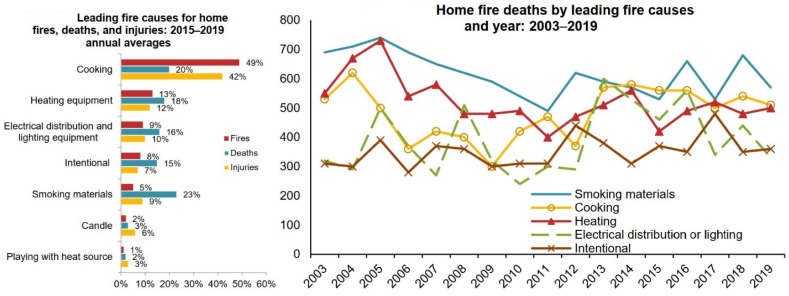
Leading causes of home fires and fire-related deaths and injuries according to 2021 NFPA report [13].

Early fire detection is a challenging yet critical problem considering its direct influence on human safety and the environment. Advanced technology requires proper approaches for detecting flames at the earliest possible moment, to prevent injury and property damage. Fire prevention and control has always been a difficult task for governments worldwide. As illustrated in Figure 1, the majority of residential fires and residential fire injuries are caused by one of five factors: cooking, heating equipment, electrical distribution and lighting equipment, purposeful fire starting, and smoking materials. According to the National Fire Protection Association (NFPA), cooking was the greatest cause of residential fires and residential fire injuries from 2015 to 2019, whereas smoking was the leading cause of residential fire deaths [13].

Traditionally, fires have been detected using sensory systems that detect changes in smoke or temperature in indoor environments. Almost all fire detection systems now have built-in sensors, and therefore the systems are essentially dependent on the sensors’ reliability and spatial dispersion. For a high-precision fire detection system, the sensors should be installed in a position that is calculated correctly and accurately. Coverage of large spaces in indoor or outdoor environments is impractical in a sensor-based fire detection system, owing to the need for a regular distribution of nearby sensors; hence, such a system has a high false alarm rate. According to the Home Office of the United Kingdom, in 2018, 38% of fire alarms failed to sound when there was a fire, and 45% of these occurrences were as a result of poor system positioning [14]. In 2020, a total number of 549,913 incidents were attended by fire and rescue services. Of these, fires accounted for 28%, fire false alarms for 42%, and non-fire incidents for 30%. Fire false alarms occur when a firefighter arrives at a site expecting to see a fire, but in fact no such occurrence exists or has existed [15]. Battery-powered detectors have a greater failure rate than mains-powered sensors, with 38 percent of battery-powered sensors failing to sound, compared to 21% of mains-powered sensors [14]. These fire false alarms present a reasonable false detection rate for comparison with vision and deep-learning-based systems. These limitations may increase, because a large portion of the world’s population and of blind people live in developing countries, where many homes do not have a fire detection system or have an expired system.

For decades, fires started by cooking and those caused by smoking materials have been the leading contributors to house fire deaths. Between 2015 and 2019, an estimated average of 179,700 fires killed 940 people, injured 5690 people, and cost USD 1.3 billion in direct property damage per year [13,16]. To prevent such fires, it is crucial to detect fires rapidly without false alarms and alert BVI people using a combination of computer vision, deep learning, and smart glasses. Furthermore, the proposed indoor fire detection system can be applied in other diverse social and industrial areas such as schools, hospitals, factories and chemical plants, offices, etc. The majority of publications concentrate on wildfires and outdoor environments for fire detection, with little or no focus on indoor environments.

To address this need, we propose a fire detection and notification system based on a novel convolutional neural network (CNN), using the YOLOv4 model [17]. Because this study is part of a whole framework, the proposed indoor fire detection system is discussed for the specific application which assists BVI people. With the advantages of smart glasses, we can improve visual information accessibility in real-world indoor situations and build a system to perform real-time fire detection tasks. The proposed system combines modern computer vision and deep learning models with wearable assistive technologies. The wearable technology is a smart-glasses-based system that converts visual input into auditory information for the assistance of the visually impaired. This was based on the smart glasses developed in our previous research [18]. In this study, we improved the performance of the classic YOLOv4 network to enable rapid detection of fire hazards and performed experiments on an AI server, as explained in Section 3 and Section 4.

In summary, the main contributions of the study are as follows:

- A fully automated fire detection and notification system was developed for BVI people, to provide fire prevention and safety information in emergencies. To the best of our knowledge, existing smart-glasses-based systems for BVI do not support indoor and outdoor fire detection. The system provides users with information regarding the fire and surrounding objects through real-time audio output.
- A large fire image dataset was created with indoor (cooking, heating, electrical equipment, smoking materials, candles, etc.) and outdoor images of fire scenes. The dataset will be made publicly available on the Internet. In a deep CNN, important features are learned using large datasets to accurately identify target information whilst avoiding overfitting. We developed a technique for automatically moving labeled bounding boxes when the fire images were flipped horizontally and vertically, for image-data augmentation.
- An object mapping approach was developed to provide the BVI with complete information about the surrounding situation and to differentiate daily lifestyle fires from hazardous fires.
- Since it uses vision-based fire detection and deep learning approaches, the proposed fire detection and notification system has several advantages over existing fire alarm systems that support only sensor-based fire detection.

The rest of the paper is arranged as follows. Section 2 describes the literature on wearable assistance systems for BVI people and indoor fire detection. The proposed fire detection and notification system is explained in Section 3. In Section 4, we present the experimental results. Section 5 discusses the limitations and future focus of the proposed system. Section 6 concludes the paper.

## 2. Related Works

Although the study of fire detection and notification applications in outdoor environments has been expanding for decades, not enough research has been applied to wearable assistive devices for assisting BVI people and to indoor situations. In this section, we review the research on wearable assistance systems for BVI people and the research on indoor fire detection. The modernization of wearable assistive systems with a vision-based fire detection model allows BVI people to improve their cognitive knowledge of the environment and make the right decisions.

### 2.1. Wearable Assistance Systems for BVI People

Smart wearable assistance systems [19,20,21,22] have rapidly become a part of everyday life in recent years, with watches, glasses, and a variety of other wearable devices being enhanced with technology. Obtaining a structural understanding of the surrounding area and executing autonomous navigation are two of the most crucial tasks for BVI individuals [23]. Aladren et al. [24] proposed a navigation system that integrated the range and color information of input images to guide BVI people around an indoor environment. The navigation system recognized and classified the scene’s primary structural aspects, allowing the user to walk securely without striking any obstacles. The key structural features of the image were detected and classified using range data. Owing to the range sensor’s limitations, color data were combined with the range data to extend floor segmentation throughout the full view. For shorter distances (up to 3 m), range data were employed, whereas for longer distances (more than 3 m), color data were used. In 2018, Xiao et al. [25] introduced a smart indoor collision avoidance system based on an RFID appliance that recognized and tracked passive RFID tags by examining the obtained backscatter signals. This resulted in a high-performance collision avoidance system for BVI people. Received signal strength indicator (RSSI) fingerprints based on the locally weighted linear regression technique were extracted to determine the distance between the user and obstacles, and the rate of unwrapped phase shifts was used to direct the user’s movement. This technology could save BVI users from striking obstacles while moving and direct them to a target area without requiring them to look around. In 2021, Barontini et al. [26] designed a wearable travel navigation system using an RGB-D camera, laptop, and a cutaneous passive haptic interface to aid BVI people in navigating unfamiliar indoor environments. An RGB-D camera was placed on the user’s chest, while a laptop was placed at the user’s waist to interpret the visual information from the camera and send orders to the clenching upper-limb force-feedback wearable device, which provided distributed tactile stimulation via normal and tangential skin forces. Recently, Plikunas et al. [27] proposed the initial outsourcing of video recordings of indoor navigation paths for blind users from an online network of seeing volunteers, who used specialized sensory equipment and web services to collect and constantly update a cloud-based database of indoor routes. These indoor navigation paths required additional input from volunteers (obtained via social networking) and AI-based computational algorithms, in order to work properly.

Recent advances in computer vision, smartphone devices, and big data technology have encouraged researchers and inventors to develop new solutions that integrate these emerging technologies to improve the mobility and overall quality of life of BVI people. Jiang et al. [20] presented a wearable assistance system based on big data and binocular vision sensors that recorded images at a predetermined frequency and selected the most useful images using stereo image quality evaluation. The ResNet network was employed for outdoor object detection tasks in this system. The NavGuide, an electronic travel assistance system that detects wet floors, floor-level barriers, and knee-level blocks, was designed and developed by Patil et al. [28]. This system classifies obstacles and their surroundings and then presents users with the most important information. In addition, the NavGuide aids BVI people in circumstances such as making a left or right turn and dealing with obstructions, and wet floors. Through tactile and aural sense-based feedback systems, NavGuide provides its user with simplified and prioritized information about the surrounding environment, using special shoes. The audio samples are saved on a micro secure digital (SD) card. The user hears audio feedback via mono wireless headphones. In 2021, Martinez-Cruz et al. [29] proposed an outdoor navigation aid system for BVI users of public transportation, which uses Bluetooth Low Energy (BLE) technology for location and communication, as well as a mobile app for user–smartphone interaction. The created smartphone application can track and determine public transportation buses and bus stops, as well as providing real-time information to users through voiced instructions. The BLE beacons in the buses and bus stations broadcast a 3 s Bluetooth signal, which is received by the mobile application, and the unique identification (ID) is retrieved. One of the most difficult tasks for a BVI person is to cross an intersection safely. To solve this problem, Shin et al. [30] presented an intersection crossing system that applies the BLE and RSSI signal concepts for indoor and outdoor position tracking to locate the BVI user successfully. It is essential to categorize the area in which blind people are located and only use the acoustic signal in that area, but it is also necessary to know the person’s real-time position, not just the area in which they are located.

A variety of solutions have been developed to help BVI individuals grasp nearby objects and make eye contact with their sighted companions. Satpute et al. [31] designed a wearable vibrotactile ring with tiny tactors (antenna-like objects) positioned along two perpendicular axes across the finger (left–right, up–down) to assist blind people in locating and reaching for objects in the space surrounding the body. A finger-mounted camera detects a target item, and vibratory signals are used to lead the user to it. At this stage, computer vision algorithms are used to identify and handle graphical controls on a computer screen, with linear and rotational motions such as virtual slide potentiometers and knobs. The social glasses-based system proposed by Qiu et al. [32] was iteratively constructed to provide multimodal feedback channels for “eye contact,” incorporating both visual and tactile inputs in gaze simulation. The system includes two components: a set of smart glasses and a tactile wristband worn by a blind user. The sensor module tracks a sighted person’s gaze and communicates the data to the Arduino board, whenever the person looks at the smart glasses. The Arduino board runs the vibration motor, which provides 1 s of tactile feedback to the blind user.

### 2.2. Indoor Fire Detection and Notification Approaches

Fire is a common everyday occurrence that poses serious hazards to human life and infrastructure, as well as causing financial damage. Indoor fires are particularly dangerous, because the poisonous emissions and smoke are trapped indoors, causing more deaths than physical burns. In recent years, various vision-based fire detection systems [33,34,35,36,37,38,39] for indoor locations have been proposed. We analyzed some of these studies. To achieve indoor safety surveillance, Chang et al. [34] designed an intelligent fire detection system that consisted of a visible human–machine interface and a remote server. This intelligent fire detection system was composed of five modules (image, infrared temperature, flame, carbon monoxide gas, and liquefied petroleum gas) and a remote server. The modules were installed in an autonomous surveillance vehicle to detect environmental sensory data, which were then sent to the server for processing. Ajith et al. [35] proposed a vision-based system for indoor or outdoor surveillance that combined spatial, temporal, and motion information to extract the fire- and smoke-containing regions from infrared video frames. Multiple characteristics such as optical flow, divergence, and intensity values are used to fuse information. Some unsupervised segmentation algorithms such as GMM, K-means, MRF, and GMRF were employed in the comparative study, and MRF performed better in the categorization, with an accuracy of 95.39%. The experimental results of [35] showed that MRF was able to differentiate fire, smoke, and background with greater precision in both qualitative and quantitative evaluations. Gagliardi et al. [36] presented a video-based smoke detection method for early warning in fire detection surveillance systems that could be used both indoors and outdoors. To extract a warning alert under a real-time deadline, the smoke detection approach used blob labeling, a Kalman estimator, image segmentation, color analysis, and geometrical feature analysis.

## 3. Materials and Methods

### 3.1. Overall Design of the Proposed System

This work aimed to increase the convenience and opportunities for BVI people when performing daily indoor activities autonomously. For this purpose, we propose a fire detection and notification system based on YOLOv4 and smart glasses, which captures images through a tiny camera and transmits them to a server equipped with an AI module that returns fire detection results with voice feedback. The proposed system uses deep CNNs to detect fire regions with high accuracy and a powerful processor to perform real-time image processing sufficiently fast. Thus, we introduce a client–server architecture consisting of smart glasses and a smartphone as the client and an AI server to perform image processing tasks.

**Figure 2:**
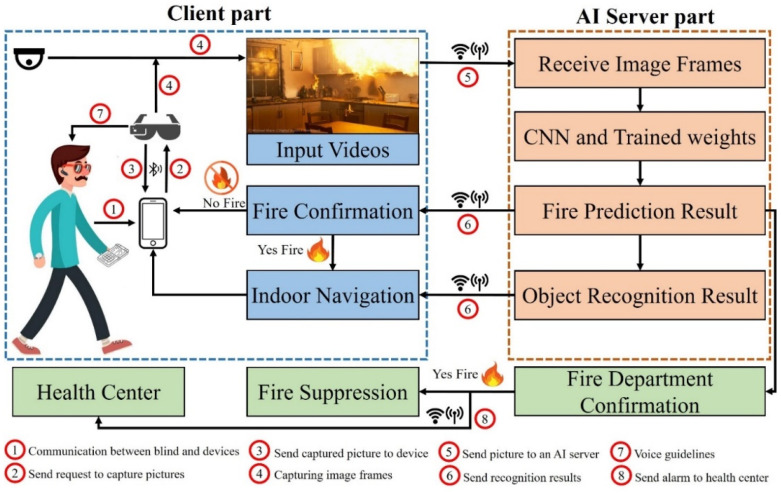
Overall design of the proposed system.

Figure 2 depicts the general architecture of the proposed system. Section 3.3 gives a more detailed explanation. We added home security cameras to deal with circumstances where BVI people are not at home, are asleep, or are not using smart glasses, preventing the early fire detection and alert system from failing. In these circumstances, the AI server sends fire prediction results to blind people and to the fire department, as shown in Figure 2. If a fire is confirmed by blind people or the fire department, it can be suppressed by activating fire extinguishing devices. The client part consists of smart glasses and a smartphone that send the data through Bluetooth and a home security camera that records continuously (continuous video recording). Meanwhile, the AI server receives the images from the client, processes them, and returns the result in an audio format. The smart glasses receive the audio results and communicate with users via the built-in speaker or through a smartphone.

The client part of the system works as follows. Initially, the user connects the smart glasses to a smartphone through Bluetooth. Subsequently, the user can request the smart glasses to capture images, which are then sent to the smartphone. The power consumption of the glasses can be reduced in this case, which is more efficient than continuous video recording. The results from the AI server are then conveyed via earphones or speakers as voice feedback. BVI users with tactile devices can also touch and feel the outline of salient objects. Despite the recent introduction of lightweight deep CNN models, we used an AI server to perform deep-learning-based computer vision tasks, because the GPUs in wearable assistive devices have limited specifications compared to a powerful AI server. Because the smart glasses and smartphones were only used for capturing photos, this extended the battery life of these devices. Furthermore, the AI server was convenient for further improving the accuracy of the deep CNN models and adding new features. Following text-to-speech, the AI server received images and applied fire detection and object recognition models to detect fires and recognize objects. Subsequently, the audio results were delivered as an AI server response to the client’s request. The results of the fire prediction are sent to the fire department for confirmation.

### 3.2. Indoor Fire Detection Dataset

The level of precision of the deep learning model primarily depended on the dataset used in the training and testing procedures. As determined by our review of datasets for fire detection, the datasets developed for vision-based fire detection systems are insufficient, and existing open-access datasets have some drawbacks. To address these issues, we created a fire image dataset for indoor fire scenes. First, we classified fires based on the material that forms the fuel source. We then researched which fires were the most common in indoor situations. To the best of our knowledge, class A and B fires represent the most common fuel sources in home fires, including wood, paper, cloth, rubber, trash, plastics, gas, and oil-based products. Finally, we gathered fire and non-fire images from various open-access sources such as Kaggle, GitHub, Google, and Flickr, finding images depicting a range of different conditions (shape, color, size, time of day, and indoor environment). Our fire image dataset consisted of 6000 indoor fire and non-fire images, as shown in Table 1.

**Figure 3:**
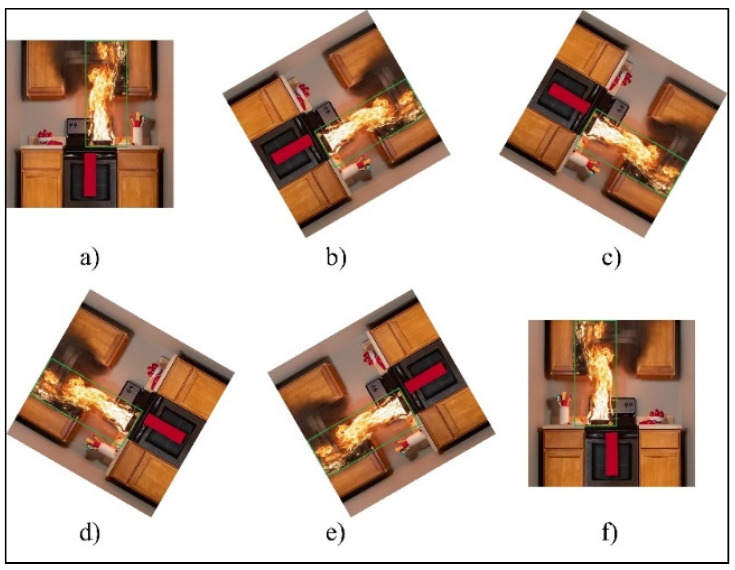
Image data augmentation using geometric transformations: (**a**) original image; (**b**) 60° rotation; (**c**) 120° rotation; (**d**) horizontal flipping of 60° rotated image; (**e**) horizontal flipping of 120° rotated image; (**f**) horizontal flipping of original image.

A large amount of labeled training data is a key factor in the success of any deep learning model. However, it was challenging to obtain robust fire detection results using this dataset in real-world scenarios. This may be due to overfitting, underfitting, or class imbalance. An overfitted model cannot capture patterns in images in an appropriate way. Underfitting can be due to a shortage of data; hence, we employed the technique of image data augmentation (modifying and reusing images) to improve the inference power of the model. After our review [40,41,42,43] and experiments [39,44], we found that image data augmentation techniques based on geometric transformations such as flipping and rotation proved to be the most effective methods for our research. The sizes of training-image datasets and their resolutions determine the power of CNN models. Therefore, we increased the number of images in the fire detection dataset by rotating each original fire image at angles of 60° and 120°, as well as horizontally flipping each original and rotated image, as shown in Figure 3. Thus, we modified the existing training images to generalize them to different circumstances, allowing the model to learn from a larger range of situations. Manually flipping, rotating, and labeling all of the images in the dataset is very time-consuming. To automate the image modification process, we designed software that can automatically flip and rotate images using the OpenCV library.

Flipping horizontally. The image was flipped along the vertical axis so that left and right exchanged positions. This is shown in mathematical terms in Equation (1), where *x* and *y* are the horizontal and vertical coordinates of the original pixel, and *Ix* and *Iy* are the corresponding elements of the resulting pixel.

(1) $$\left[\begin{array}{c} I_{x}\\ I_{y} \end{array}\right]=\left[\begin{array}{cc} -1 & 0\\ 0 & 1 \end{array}\right]\cdot \left[\begin{array}{c} x\\ y \end{array}\right]$$

Flipping vertically. Similarly, this means flipping about the horizontal axis, interchanging the top and bottom of the image, as shown in Equation (2):

(2) $$\left[\begin{array}{c} I_{x}\\ I_{y} \end{array}\right]=\left[\begin{array}{cc} 1 & 0\\ 0 & -1 \end{array}\right]\cdot \left[\begin{array}{c} x\\ y \end{array}\right]$$

Rotation of the image. The analogous rotation is given in Equation (3):

(3) $$\left[\begin{array}{c} I_{x}\\ I_{y} \end{array}\right]=\left[\begin{array}{cc} cos\;\varphi & -sin\;\varphi \\ sin\;\varphi & cos\;\varphi \end{array}\right]\cdot \left[\begin{array}{c} x\\ y \end{array}\right]$$

The coordinates of the flame in the image naturally change when the labeled pictures are rotated at specific angles. To avoid labeling them again manually, we read all of the pictures in the folder, transformed them into angles, and developed special software to update their labels. Using the LabelImg tool 1.8.0, we modified the location of the fires in each picture according to the YOLOv4 training annotation. The tag folder was a text file that tracked the fire coordinates. It was also used as part of the learning process in a CNN. We also utilized non-fire and fire-like images in the training set, to decrease false-positive detections.

The 6000 fire detection images were divided into training and test sets, with 80 percent (4800) used for training. We enlarged the dataset images by five times the number of original augmented images after using data augmentation methods on only the training set (Figure 3). As shown in Table 2, the total number of fire detection images increased to 30,000.

### 3.3. Implementation of Fire Detection and Notification System

The overall design of the proposed system, which included client and AI server components, is explained in Section 3.1. In this section, we explain the processes of deep-learning-based computer vision techniques that run on an AI server. In our approach, several computer vision techniques using deep learning were developed to achieve our goals.

**Figure 4:**
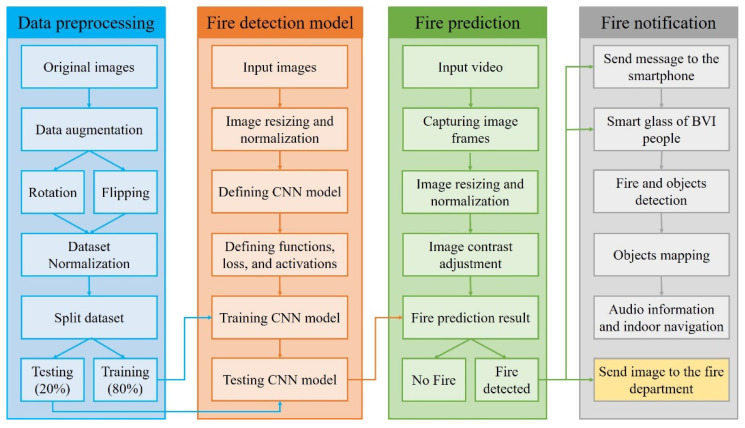
Block diagram of the proposed system.

Data preprocessing. As illustrated in Figure 4 (and explained in Section 3.2), we first collected 6000 images to create an indoor fire detection dataset. Then, we increased the indoor fire detection accuracy using the fire image dataset and improved the deep CNN model. Currently, the YOLOv4 model is one of the most suitable deep CNN models for training with a custom image resolution. The image resolution for the YOLO model must be a multiple of 32. Therefore, we resized the original fire images in the dataset to a standard resolution of 416 × 416 pixels, because the training process took more time than expected with large input images and low frames-per-second (fps) values [43]. However, the performance of the trained model was observed at different image resolutions at the test stage, including 416 × 416, 512 × 512, 608 × 608, 832 × 832, and 960 × 960.

We performed quantitative experiments by applying object detection evaluation metrics, including precision, recall, and average precision (AP), as in our previous research [18,44,45,46], and analyzed the results. Precision is the ability of a classifier to identify only the relevant objects, i.e., the proportion of true positives detected. Recall measures the ability of the model to identify all relevant cases; it is the proportion of true positives detected among all ground truths. A good model is one that can identify most ground-truth objects (it exhibits high recall) while identifying only the relevant objects (it exhibits high precision). A perfect model has a false-negative value of 0 (recall = 1) and a false-positive value of 0 (precision = 1). Precision and recall rates were obtained by comparing pixel-level ground-truth images with the results of the proposed method. We used the following equations to calculate the precision and recall metrics of indoor fire detection systems:

(4) $$Precision_{C_{ij}}=\frac{TP_{C_{ij}}}{TP_{C_{ij}}+FP_{C_{ij}}},$$

(5) $$Recall_{C_{ij}}=\frac{TP_{C_{ij}}}{TP_{C_{ij}}+FN_{C_{ij}}},$$

where *TP* denotes true positives and is the number of correctly detected fires, *FP* denotes false positives and is the number of background regions detected as fires, and *FN* denotes false negatives and is the number of fires detected as background regions. We calculated the average precision (AP) as shown in Equation (6):

(6) $$AP_{C_{ij}}=\frac{1}{m}\sum_{j=1}^{m}Precision_{C_{ij}},$$

**Figure 5:**
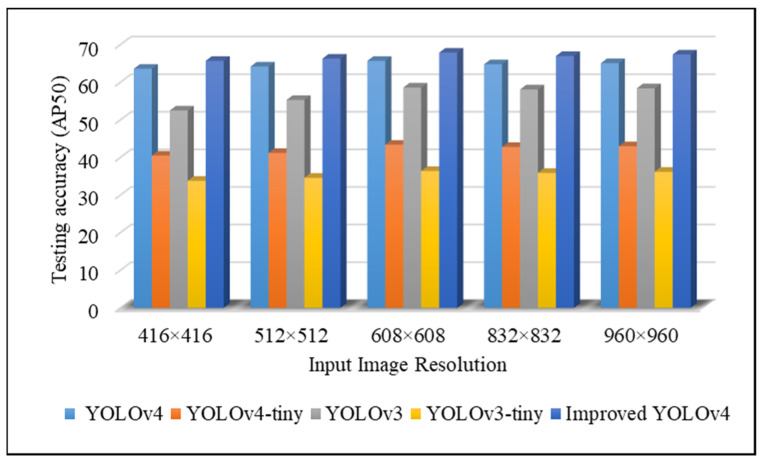
Performance of different systems at various input image resolution settings at testing stage with original images.

Figure 5 shows that the performance of the model improved with increasing size of the test images, and the best performance was at 608 × 608 pixels.

Next, we tested the performance of the deep CNN model with the original 6000 images, then with the full augmented dataset. The performance of the deep CNN model was better with the full dataset than with the original dataset, as shown in Table 3.

**Figure 6:**
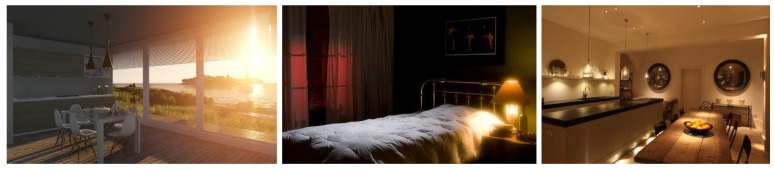
Example of non-fire images in our dataset.

After completing the training and testing procedures, we tested 1876 daytime and night-time pictures that were similar to the fire scenes. The number of false positives from these 1876 images also assisted in checking the performance of the trained weights. Sunlight is a common distraction for fire detection cameras; therefore, we included non-fire and fire-like images including sunsets, sunrises, and lighting in our dataset. In this research, we used 1876 non-fire and fire-like images such as sunrises, sunsets, and lighting in the training and testing steps of the model. This is because sunlight and lighting pixel values are very close to fire color intensities, even though they are not actual fires. Examples of non-fire images are shown in Figure 6.

Fire detection model. YOLO detection is an object classification system based on AI. YOLO has been released in five versions thus far: YOLOv1 through YOLOv5. Currently, almost all versions are used to identify objects, although not all series are equally effective in detecting fires. In this study, we used YOLOv4, which is an extension of the YOLOv3 model, for the fire detection task. YOLOv4 is a real-time, high-precision, single-stage, regression-based object detection model which was presented in 2020. It incorporates the features of a series of YOLO detectors such as a path aggregation network (PANet), Mish activation function, spatial pyramid pooling (SPP), self-adversarial training, mosaic data enhancement, CmBN, and many other techniques to significantly enhance detection precision. The model structure consists of three parts: feature extraction (CSPDarknet53), the feature fusion or neck (PAN and SPP), and prediction (bounding box). We made some improvements to the original YOLOv4 network architecture to obtain robust indoor fire detection results. To reduce the running time and increase the robustness of the deep CNN model, the h-swish activation function was used to ensure elimination of gradient explosion. Other parts of the deep CNN model were improved by adding a convolutional block attention module, as presented in [47]. We tested the performance of the proposed approach by experimenting with other versions of YOLO on the original fire dataset (6000 images) and compared the final precisions (Table 4).

Fire prediction. At the fire prediction stage, smart glasses and security cameras record video and capture image frames, which are then sent to the AI server for processing. The AI server receives and resizes the image frames to a 608 × 608 resolution. Usually, in indoor settings, the contrast of the images is low, owing to the lack of natural light and other external factors; therefore, we applied contrast enhancement methods to the input images to obtain the desired results. In pixel transformations, the value of each output pixel depends only on the values of the corresponding input pixels. Brightness and contrast are reasonable examples of pixel modifications that increase image quality.

(7) O(x)=α I(x)+β

**Figure 7:**
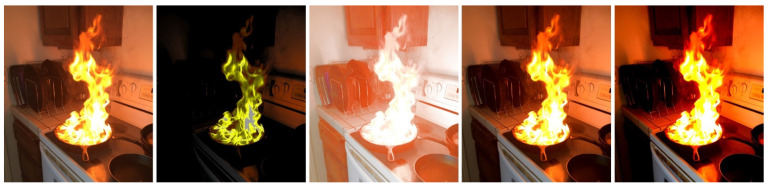
Example of fire images after contrast and brightness adjustment.

In Equation (7), *α* > 0 and *β* are the gain and bias parameters, respectively. These parameters affect image contrast and brightness. *I*(*x*) represents the source pixel of the original image and *O*(*x*) denotes the output pixel of the final image. To make Equation (7) easier to understand, we consider Equation (8):

(8) O(i,j)=α I(i,j)+β

where *i* and *j* denote the pixel in the *i*-th row and *j*-th column. By modifying the weights of *α* (contrast [1,2]) and *β* (brightness [10,11,12,13,14,15,16,17,18,19,20,21,22,23,24,25,26,27,28,29,30,31,32,33,34,35,36,37,38,39,40,41,42,43,44,45,46,47,48,49,50]), we generated an augmented image in the dataset. Brightness enhancement is one of the most effective approaches for image refinement during preprocessing. We experimentally tested these techniques in our previous research [39,44], using global color contrast enhancement [46,49], and combined local and global contrast enhancement approaches, as shown in Figure 7.

In addition, we tested the performance of the proposed approach by experimenting with other versions of YOLO on the augmented fire dataset (30,000 images) and compared the final precision results. Table 5 shows that the improved YOLOv4 model ranked the highest in the training and testing stages, with 73.6% and 71.5% accuracy, respectively. In addition, YOLOv4 achieved 72.8% (a difference of 0.8% from the improved YOLOv4 model) in testing, only marginally behind the improved YOLOv4 model in terms of testing accuracy. In training, YOLOv4-tiny and YOLOv3-tiny reached accuracies of 51.5% and 43.9%, respectively. Due to the larger quantity of dataset images, these models took longer than those in previous experiments. Although the processing time was more than that of the YOLOv4-tiny method, YOLOv4 was regarded as an efficient and strong fire detection model with the highest prediction accuracy. Using data augmentation methods, we increased the training accuracy from 69.3% to 73.6% (4.3%) and the test accuracy from 67.9% to 71.5% (3.6%).

**Figure 8:**
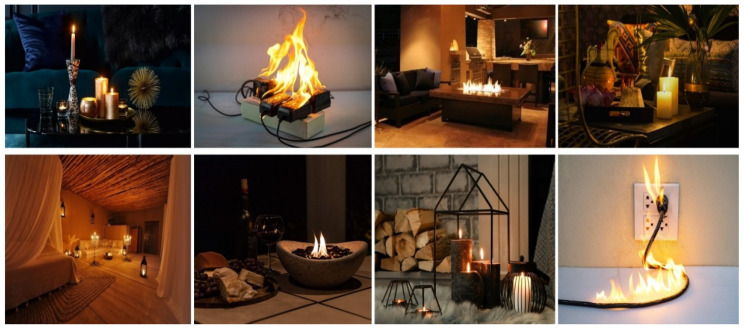
Examples of small fire images for indoor fire detection dataset.

Although we obtained 71.5% accuracy with the test set, we further researched and assessed numerous recently presented methods to enhance this result. To the best of our knowledge, most proposed methods fail in small-sized fire image detection [50]. Thus, we gathered small-sized fire images to increase our dataset and enhance the fire detection accuracy. Figure 8 shows some examples of small-sized fire images. As indicated in [44], we used a large-scale feature map to detect tiny moving objects and concatenated it with a feature map from prior layers to maintain the fine-grained features. This large-scale feature map was used to identify small-sized fire pixels by combining the location information from earlier layers with complicated characteristics from deeper levels. We improved the fire detection accuracy to 72.6% using the test set.

Fire notification. Once fire regions are detected, two different actions are triggered for the fire notification stage: (1) the AI server sends audio and text messages to the user’s smartphone, and (2) the AI server sends a detected fire image to the fire department. Regarding the first action, BVI people can control the surrounding situation and differentiate daily lifestyle fires from hazardous fires by wearing smart glasses. If they confirm a hazardous fire, they can self-evacuate using fire, object, and text recognition [51] methods, with object mapping methods to determine the relationships among different objects. The relationships among objects can provide additional information using keywords including “in”, “on”, “next to”, “below”, and “above”, for instance, “fire above oven” or “fire next to chair”. BVI people can hear voice guidelines and receive tactile information to assist indoor navigation from fire zones to safe zones. The most challenging relationships relate to the keywords “on” and “in” because they rely on the interaction of an object’s pixels with another object’s top line. If two bounding boxes are within a defined range of pixels (pixel tolerance) from one another when testing horizontally, the “next to” relationship is specified. Because separate objects might have appendages, the “below” and “above” relationships are specified by using the mass boxes of the objects rather than the bounding boxes. The bounding box for a table object, for example, would contain the table’s legs and be centered in the free area underneath the table. The mass box is centered closer to the table’s real surface. The mass box for an object is specified by beginning with the assigned coordinates of the bounding box and checking the image one axis at a time. The total number of pixels of the mask remaining in the box are multiplied by the percentage of pixels of the mask remaining in the box at each iteration of the pixel movement. The “below” and “above” keywords examine how closely the pixels from each object are aligned with each other, using the center of the mass box of the object.

Regarding the second action, the fire department receive a photo of the fire and can determine whether it is a daily lifestyle fire or a dangerous fire. If it is a potentially dangerous fire, they can follow the fire department’s procedures.

## 4. Experimental Results

In this section, we describe the experimental setup and the results of the fire detection models on the AI server. We trained the proposed deep CNN model on a PC with an 8-core 3.70 GHz CPU, 32 GB RAM, and NVidia GeForce 1080Ti GPUs. Experimental validations of the proposed wearable assistive fire detection and notification system were conducted in an indoor environment, with a focus on fire detection. We used our indoor fire detection dataset for training and testing. The key parameters for the training experiments were: width and height of the input images, 416 pixels; batch size, 32; subdivision, 8; learning rate, 0.01.

Using a high-performance AI server is more effective than using embedded systems to increase the energy storage viability of smart glasses and ensure real-time system performance [18]. The performance of the AI server specifies whether the proposed wearable assistive fire detection system succeeds or fails. This is because deep learning models for fire detection and notification systems consume a significant amount of computing resources on an AI server. Thus, to evaluate the performance of the proposed system, we conducted experiments using an AI server with a powerful specification, as shown in Table 6.

First, the client part transfers images to the AI server for image processing. Then, the AI server processes the received images using the proposed fire detection module. Thereafter, the fire detection results are converted to the audio format using a text-to-speech module and sent back to the client through an Internet connection. Finally, the audio results are played via a speaker or earphones. If BVI users have a refreshable tactile display, they can touch and sense the contours of detected objects [52]. The qualitative and quantitative evaluations of the fire detection module on the AI server are presented in the following subsections. We used a Raspberry Pi 3 Model B+ attached to regular glasses to make a prototype of the smart glasses, as specified in Table 7.

The 8 MP device camera was used to take video and photographs, since it is capable of taking high-resolution pictures and full HD 1080p video and is entirely programmable.

### 4.1. Qualitative Evaluation

**Figure 9:**
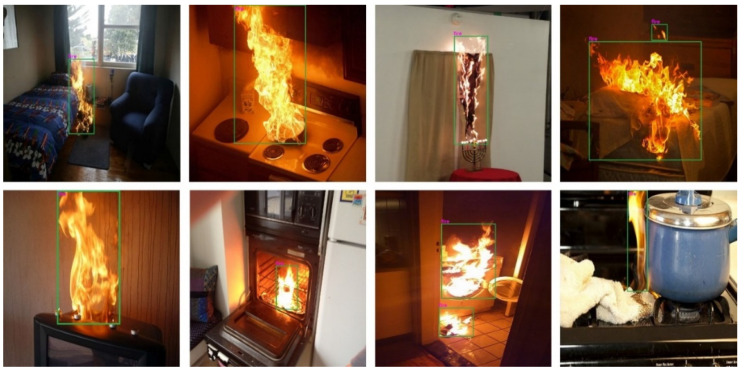
Visual results of the proposed fire detection system for various indoor environments.

Initially, we evaluated the proposed fire detection system qualitatively. For this purpose, we randomly selected eight fire images from the test set of our fire detection dataset. The qualitative results of the improved YOLOv4 model for the eight images are shown in Figure 9. These eight images have various environments and contexts, such as fires resulting from cooking or heating appliances, electrical fires, and living room fires.

**Figure 10:**
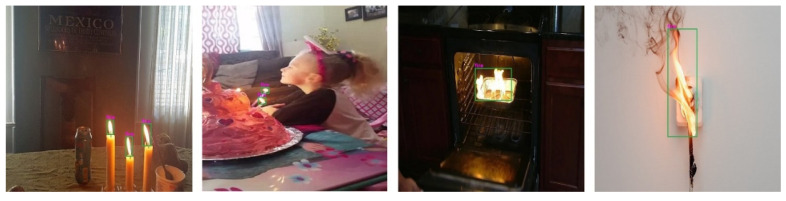
Visual results of the proposed fire detection system for different small-sized fire scenes.

As shown in Figure 9, the proposed fire detection system using the improved YOLOv4 model accurately detected fires in different indoor environments. It can be added as a configurable module to smart glasses [18] to help BVI people identify fires in their surroundings. We also experimented with small-sized fire images to check the robustness and reliability of the proposed method. Early fire detection is critical in fire prevention and suppression. Small fires can damage objects in a short period of time, for example 10–20 s, and even cause harm to human health and life. Some examples of fire detection results for small-sized fire images are shown in Figure 10.

**Figure 11:**
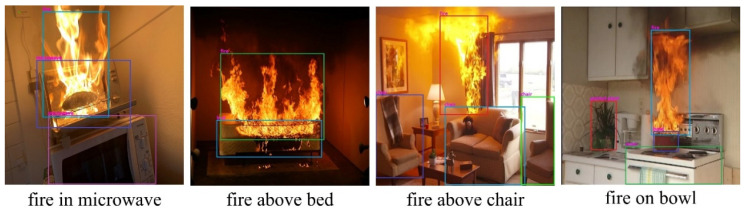
Example results of object mapping for image annotation.

The proposed fire detection system was also able to correctly identify small fire regions, as shown in Figure 10. In real life, however, people use fire while performing daily activities such as cooking meals using a gas oven, lighting candles on cakes, and using electric fireplaces. It is extremely difficult to distinguish daily intentional fires from hazardous ones. Therefore, we proposed an object mapping method to determine the relationships among objects. We used keywords such as “in”, “on”, “below”, “above”, and “next to” for extra information. The experimental results for the relationship between the object and the fire are shown in Figure 11. In this experiment, we used various indoor fire images from the test set of our dataset. Thus, the experimental results showed that the fire and object detection models performed accurately with the object mapping method. Object detection and mapping worked effectively, even when multiple objects were present, as shown in Figure 11. The data for the recognized objects and the relationships between these objects were converted to audio and sent to the client through the network. In addition, as explained in Section 3, the detected fire image was sent to the fire department simultaneously for confirmation.

Experiments have shown that our proposed method may reduce blind users’ fears and allow for early suppression and fast response, regardless of the time of day or the size or shape of the fire. Traditional visual fire detectors generate false alerts when the color and pixel intensity values of objects are similar to those of fires, particularly for small fires.

### 4.2. Quantitative Evaluation

We performed quantitative experiments by applying object detection evaluation metrics, including precision, recall, and AP, as calculated in Equations (4)–(6).

Table 8 shows the performance comparison between the improved YOLOv4 model and other popular object detector models such as CVPR, ECCV, and ICCV that have been published in top journals and conferences in recent years. We used the same training and testing fire images from the custom fire dataset to compare and evaluate the performances of the object detector models.

As we can see, the improved YOLOv4 model achieved the best fire detection performance on our fire dataset in terms of the AP, AP50, AP75, APM, and APL evaluation metrics. The proposed method achieved the second-best overall performance, being slightly inferior to the original YOLOv4 only in terms of the APS evaluation metrics.

### 4.3. Analysis of Fire Detection Systems Based on Thermal, Smoke, and Vision Sensors

*Thermal Sensors.* Heat is a type of thermal energy that flows from a hot area to a cooler area. Heat sensing utilizes a heating component or an infrared camera to detect the amount of thermal energy that is transported by convection. The heating component detects temperature changes caused by changes in refractive index, displacement, resistance, and other factors. A thermal sensor mainly consists of three parts: signal conditioning, an amplification circuit, and a heating component [56]. The thermal sensor is utilized to determine the amount of heat present in an indoor environment due to fires. Fixed temperature, rate of increase, and rate of compensation are the three types of thermal sensors available. The thermal sensor uses a minimum working temperature or a specified temperature threshold. When the air temperature exceeds the predetermined temperature, the rate of compensation thermal sensor is activated.

*Smoke sensors.* The most common and widely used fire alarm systems nowadays are based on smoke sensors. Photoelectric detectors (light scattering) and ionization detectors are two approaches for detecting smoke for fire detection. An ionization smoke sensor employs a radioactive source, whereas photoelectric sensors comprise a photodetector and a light emitter. If there is smoke in the room, the smoke particles disperse light. The detector is used to measure light distribution or obscuration. Normally, the fire alarm signal is activated when the signals surpass a predetermined threshold, regardless of the detecting principle. The sensing principle determines the reaction time, reliability, and sensitivity of the fire alarm. Usually, ionization alarms respond faster than photoelectric alarms to open fires with flames. In contrast, photoelectric alarms tend to show faster response and higher sensitivity than ionization detectors for smoldering fires [56]. Briefly, smoke detectors can be considered as particle detectors that are sensitive to a specific distribution of particle sizes. Typically, a fire alarm is activated when a smoke sensor signal exceeds a predetermined threshold. As a result, when the particles have comparable sizes or refractive indices, these systems can fail to distinguish between fire-related and non-fire-related particles. For example, smoke detectors are sensitive to dust and moisture. Furthermore, they are unable to distinguish between combustion outcomes created under regulated circumstances such as cigarette smoke or some cooking processes and combustion products produced in a dangerous fire environment. Finally, cross-sensitivities exist in both photoelectric and ionization fire alarm systems, resulting in a high false alarm rate. Sometimes, the false alarm percentage becomes too high, so that some householders or business owners are willing to disable or ignore fire alarm signals. To reduce false fire alarm signals and increase the accuracy of fire detection, other sensors can be added to smoke sensors.

*Vision-based fire detection.* The issue with traditional heat, smoke, flame, and gas sensors is that they require an excessive amount of time to reach their target values. This is the time required for particles to reach and activate the point sensors. Another concern is the minimal coverage area. As a result, many point sensors are necessary to cover large areas. A fire is characterized by its location, color, size, shape, development, dynamic texture, and degree of burning. Conventional sensors are incapable of detecting all of these nuanced features. The majority of conventional sensors create false alarm signals and incur extra economic costs. These issues can be reduced significantly by using cameras to capture and evaluate fire images. Additionally, surveillance cameras can be used in place of dedicated fire detection cameras, in order to save money.

*Economic Costs of False Fire Alarms.* According to Fire and Emergency New Zealand, more than 25,000 false alarms are attended every year. For individual fire departments, between 30 and 90% of all calls turn out to be false fire alarms [57]. These false fire alarms occur due to the detection of smoke, heat, airborne contaminants, occupancy activity, or fire alarm system faults rather than resulting from an actual fire. False alarms impose a variety of expenses on emergency services, the general public, residents, and building owners. Business interruptions can be costly, particularly if production lines must be shut down and restarted, inventory is spoiled, or deadlines are missed. In addition, there are a variety of other expenses associated with traffic accidents, professional firefighters’ job satisfaction, congestion, and adverse effects on volunteer firefighters, their families, and businesses. Several reports and research initiatives in the United Kingdom, the United States of America, and Australia have been examined to acquire knowledge on worldwide methods for reducing false alarms and their application. The reports mainly identify six solutions for reducing false alarms, listed here in order of effectiveness. The first is replacing individual smoke or heat detectors with intelligent multi-sensor detectors, with the significant potential to reduce false alarms by 69% [57,58]. The solutions are:

1. Replace detector with multi-sensor detector.
2. Use of appropriate approved detectors located correctly.
3. Use of cameras where required.
4. Communication and cooperation between fire departments and the alarm industry.
5. Better control of contractors.
6. Better data collection and national databases to enable proper analysis of the causes and patterns of false alarms.

Additionally, we acquired the frame processing time performance for each stage of the proposed system, including Bluetooth image transmission between the smart glasses and smartphone, 5G/Wi-Fi image transmission between the smartphone and server, and image processing time for the fire detection and notification module in the AI server. The average processing time for each stage is shown in Table 9. As can be seen, the overall duration for all steps was 1.32 s, which is acceptable in real-world settings.

## 5. Limitations and Future Research

**Figure 12:**
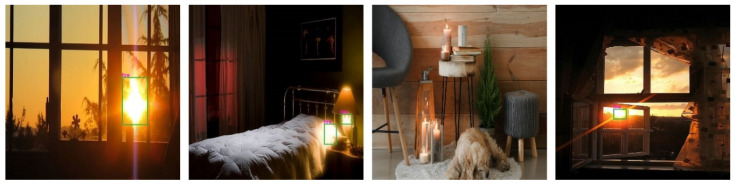
Experimental results showing limitations of the proposed fire detection system.

Despite the aforementioned achievements, the proposed fire detection and notification system has certain shortcomings. These include detecting small fire regions and distinguishing real fires from artificial fires or fire-like situations such as sunsets, sunshine, lighting, and electric lamps. Figure 12 illustrates these drawbacks. These limitations mainly occur in dark scenes, where the pixel values of the sunshine or lighting are very similar to the pixel values of a fire. Furthermore, it is also necessary to improve the indoor navigation system so that blind users can evacuate themselves from the danger zone once a fire is detected. To do this, our next aim is to first increase the number of object classes in the dataset and then update the smart-glasses-based system using an RGB-D camera or ultrasound sensor that detects the distance to the object. Adding a method to determine the size of the fire and how far it is from a blind person is also one of the tasks that could expand the scope of this field. Note that this study covered only the AI server part of the wearable assistive fire detection and notification system and the hardware perspective, which is the client part of the system. A case study with BVI people could not be performed owing to device patenting, the pandemic, and other circumstances. In addition, the current research analysis indicates that it is challenging to detect fires at an early stage using vision-based fire detection approaches.

## 6. Conclusions

In this research, a fire detection and notification system was developed for BVI people using deep CNN models and an improved YOLOv4 object detector. The proposed fire detection system was trained using a custom indoor fire image dataset that contained different indoor fire scenes and ran on an AI server. It detected fire regions and other surrounding objects to assist BVI people in performing daily home tasks and self-evacuating when a fire occurs in an indoor environment. We created a fire detection dataset that included 6000 fire and non-fire images for model training and testing. Furthermore, we added an object mapping method to provide BVI people with relationship information between objects and the fire. This information is helpful for BVI individuals in differentiating daily lifestyle fires from hazardous fires and navigating during self-evacuation. During the experiments, we evaluated the qualitative and quantitative performance of the proposed system by comparing it with other well-known one-stage object detectors. The experimental results and evaluation proved that the improved YOLOv4 model was robust and performed slightly better than YOLOv4 on our fire detection dataset, with 48.6 and 47.2% AP, respectively. The proposed fire detection and notification method is effective and can be used in various applications, allowing researchers to detect fires at an early stage.

## Figures and Tables

**Table 1:** Image sources for fire detection dataset.

Dataset	Number of Fire Images (4124)				Number of Non-Fire Images (1876)				Total
	Kaggle	GitHub	Google	Flickr	Kaggle	GitHub	Google	Flickr	
Indoor fire detection	1356	1584	621	563	853	624	216	183	6000

**Table 2:** Distribution of training and testing images in the indoor fire detection dataset.

Indoor Fire Detection Dataset	Number of Training Images			Number of Testing Images	Total
	Original Images	Image Rotation	Image Flipping	Original Images	
Fire images	3328	6656	9984	796	20,764
Non-fire images	1472	2944	4416	404	9236
Total	4800	9600	14,400	1200	30,000

**Table 3:** Training accuracy before and after data augmentation (DA).

Dataset	Input Size	Training (AP_50_)		Training Time		Weight Size	
		Before DA	After DA	Before DA	After DA	Before DA	After DA
Improved YOLOv4	416 × 416	69.3%	73.6%	74 h	97 h	268 MB	316 MB

**Table 4:** Accuracy of models with original fire images (6000 images).

Models	Training Input Size	Training (AP_50_)	Testing Input Size	Testing (AP_50_)	Training Time	Iteration Number
YOLOv4 [17]	416 × 416	68.5%	608 × 608	65.7%	78 h	150
YOLOv4-tiny [17]		46.2%		43.4%	9 h	
YOLOv3 [48]		61.8%		58.6%	86 h	
YOLOv3-tiny [48]		40.2%		36.4%	12 h	
Improved YOLOv4		69.3%		67.9%	74 h	

**Table 5:** Accuracy performance with augmented dataset (30,000 images).

Models	Training Input Size	Training (AP_50_)	Testing Input Size	Testing (AP_50_)	Training Time	Iteration Number
YOLOv4 [17]	416 × 416	72.8%	608 × 608	70.3%	102 h	900
YOLOv4-tiny [17]		51.5%		48.4%	12 h	
YOLOv3 [48]		68.2%		63.1%	110 h	
YOLOv3-tiny [48]		43.9%		40.6%	15 h	
Improved YOLOv4		73.6%		71.5%	97 h	

**Table 6:** The detailed specifications of the AI server.

Hardware Parts	Detailed Specifications
Graphic Processing Unit	GeForce RTX 2080 Ti 11 GB (2 are installed)
Central Processing Unit	Intel Core 9 Gen i7-9700k (4.90 GHz)
Random Access Memory	DDR4 16 GB (4 are installed)
Storage	SSD: 512 GB HDD: 2 TB (2 are installed)
Motherboard	ASUS PRIME Z390-A
Operating System	Ubuntu Desktop
Local Area Network	Internal port—10/100 Mbps External port—10/100 Mbps
Power	1000 W (+12 V Single Rail)

**Table 7:** The detailed specifications of the smart glasses prototype.

Hardware Parts	Detailed Specifications
Processor	Broadcom BCM2837B0 chipset, 1.4 GHz Quad-Core ARM Cortex-A53 (64 Bit)
Graphic Processing Unit	Dual Core Video Core IV^®^ Multimedia Co-Processor
Memory	1 GB LPDDR2 SDRAM
Connectivity Wireless LAN	2.4 GHz and 5 GHz IEEE 802.11.b/g/n/ac, maximum range of 100 m.
Connectivity Bluetooth	IEEE 802.15 Bluetooth 4.2, BLE, maximum range of 50 m.
Connectivity Ethernet	Gigabit Ethernet over USB 2.0 (maximum throughput 300 Mbps)
Video and Audio Output	1 × full size HDMI, Audio Output 3.5 mm jack, 4 × USB 2.0 ports
Camera	15-pin MIPI Camera Serial Interface (CSI-2)
Operating System	Boots from Micro SD card, running a version of the Linux operating system or Windows 10 IoT
SD Card Support	Micro SD format for loading operating system and data storage
Power	5 V/2.5 A DC via micro USB connector

**Table 8:** Accuracy performance with augmented dataset (30,000 images).

Model	Backbone	Image Size	AP	AP_50_	AP_75_	AP_S_	AP_M_	AP_L_
YOLOv3 [48]	Darknet-53	608 × 608	39.6%	63.1%	40.3%	24.7%	40.3%	44.6%
YOLOv3-tiny [48]	Darknet-53		21.7%	40.6%	23.5%	11.4%	24.7%	29.5%
RFBNet [53]	VGG-16		40.3%	61.9%	42.6%	23.1%	43.4%	49.7%
SSD [54]	VGG-16		36.8%	56.5%	37.1%	18.3%	37.8%	45.2%
RefineDet [55]	VGG-16		41.6%	61.4%	41.9%	22.6%	43.2%	45.8%
YOLOv4 [17]	CSPDarknet-53		47.2%	70.3%	52.4%	**31.8%**	50.1%	56.4%
YOLOv4-tiny [17]	CSPDarknet-53		26.4%	48.4%	29.6%	15.2%	29.3%	34.5%
Improved YOLOv4	CSPDarknet-53		**48.6%**	**72.6%**	**53.7%**	31.3%	**51.5%**	**58.1%**

**Table 9:** Average frame processing time (in seconds) for each sequence.

Transmission and Image Processing	Average Frame Processing Time (s)
Bluetooth transmission	0.12
5G/Wi-Fi transmission	0.34
Fire detection and notification module	0.86
Total	**1.32**

## Data Availability

Data sharing not applicable.
